# The Treatment of Bright’s Disease

**Published:** 1883-12

**Authors:** Charles W. Purdy

**Affiliations:** 68 Madison Street


CHICAGO MEDICAL

JOURNAL & EXAMINER.

VOL. XLVII.—DECEMBER, 1883.—NO. 6.

### Original Communications.

ARTICLE I.

THE TREATMENT OF BRIGHT'S DISEASE.—By CHARLES W. PURDY, M.D.

Bearing in mind that the chief function of the kidney is the removal of nitrogenous waste from the system, it is possible to lay down a few general rules for management of the whole class of diseases ranged under the term of albuminuria, especially when they reach a point in their progress which seriously embarrasses the function of the organ. It may be useful first to point out a few of these general measures applicable to the whole class, before proceeding to deal with each lesion in particular and its special indications.

First in importance, then, may be reckoned the diet of the albuminuric patient. It was formerly held, and is still held by some, that the loss of albumen represented a waste of tissue that must be made up by the boldest resort to nitrogenous diet, else the general condition of the patient would sink, and exhaustion become a grave complication of the case.

Such doctrine is a very dangerous one to teach, and much more so to practice,above all in nephritis. In the first place, it is a fact beyond dispute that the quantity of albumen contained in an ordinary repast of food is equal to the loss by the kidneys in ordinary cases for a week ; hence the loss of albumen by the kidneys unless very extreme, does not cause the alarming waste that many are led to suppose. The rapid anæmia of Bright's disease is not alone due to waste of albumen, but to the overcharging of the blood with tissue waste which renders that fluid unfit for healthy nourishment. The pernicious effect of still further loading the blood with highly nitrogenized foods not only increases the evils it is intended to remedy, but it is pretty sure to lead to the most dangerous of complications, namely, that of uræmia. It must never be lost sight of in the treatment of damaged kidneys, the important part always played in the retrograde metamorphosis of tissue, especially the nitrogenized principles in their relation to the urine. Fothergill remarks that "these nitrogenized matters do not merely go towards tissue formation, and then by a process of oxidation pass from one form of hystolitic product to another. They do not break up in tissue disintegration into creatin creatinin, tyrosin and other early products of tissue decay and then pass on into uric acid and urea. Besides being in themselves powerful narcotic poisons,causing coma and convulsions they also form within the animal organism ferments, which exercise no unimportant action.

The kidneys being the chief means of removing nitrogenized waste, it is clear that the presence of unusual quantities of waste nitrogen in the blood maintains these organs in a highly active functional state, and hyperæmia of functional activity leads with certainty to tissue change. Nor, indeed, is it essential in these cases to introduce large quantities of nitrogenous foods into the system, even with the view of repairing tissue waste. A very small portion is necessary for such purpose, especially if the patient is not taking much active exercise (which he should not do if suffering from albuminuria). We all take vastly more nitrogenous food than is necessary for tissue repair. Note, for instance, the robustness of the highlander, whose condition of health will compare favorably with any race of men with which we are acquainted, yet his food consists for the most part of hydrocarbons, the nitrogenous forming an insignificant part.

We now know that urea is formed not alone from disintergrating tissue, but also largely from splitting up of albuminoids in the liver, and it is time that we applied to the therapeutics of renal disease the lesson which physiology teaches.

We would suggest as a diet for the albuminuric patient as follows: In the main it should consist of farinaceous articles, fish, vegetables, and fruits. Meats must be indulged in sparingly; very small quantities of lean meat alone being permissible. Soups should be prohibited, even the conventional beef-tea and beef extracts. Eggs should be excluded from the diet in albuminuria. It has been shown by Lehmann and Stockvis that when the white of egg is introduced into the circulation, not only does that escape by the kidneys, but a surplus of other albuminoids accompanies it. Senator says the lesson will apply to meat as well as eggs. "Any excess acts in two ways injuriously—by increasing unnecessarily the amount of urea and other waste products in the blood; and also by pouring into the system an overplus of peptones or other albuminous matters, which may simply have to be excreted and cause irritation in the act." Cheese acts in a similar manner, and should not be used. Vegetables may be used freely, and the only ones to be avoided are the leguminous ones which are too rich in albumen. Fats may be used as freely as the condition of the stomach will permit. Milk is one of the best articles of diet, but should not be too exclusive as it does not furnish the elements of diet in a suitable proportion. The stomach should not be overloaded, it being an occasional observation that even in healthy persons albumen appears in the urine after a large meal. Small meals, more frequently repeated than usual, is a good rule to follow in such cases. Great discrimination is necessary in the matter of drinks in Bright's disease. Alcohol in large quantites, especially in concentrated form, is generally believed to be injurious. If alcohol be permitted at all it must be well diluted, and it is preferable to give it with some alkali or neutral water, as Vichy or Apollinaris water in excess. Alcohol stimulates the interstitial changes in the kidneys if used in quantities, hence the allowance should be very small—not enough to disturb to any extent the general circulation. Claret, Sherry and Marsala are the least objectionable. As to malt liqors, they should, as a rule, be excluded, though it is stated that lighter pale ales or Bavarian beer are nearly free from objectionable qualities.

Next in importance to diet in the management of albuminuria, as a general measure, may be reckoned the clothing and care of the skin. The cutaneous system performs a compensatory office to impeded renal action, of no small importance. Urea, we know, is constantly present in the cutaneous secretions, as are also phosphates, chlorides, and sulphates of the alkalies. Funke estimates that 157½ grains of urea pass off in perspiration in 24 hours, which is equal to one-third the amount excreted by the kidneys themselves. The same observer reckons the fluids eliminated by the skin to vary from 2 to 29 ounces per hour, and solids for the same time from 14 to 107 grains. Now, the skin, in cases of albuminuria, unfortunately, for the most part is dry and inactive. The beneficial effects of establishing a free diaphoresis in acute nephritis, are very well known to all who have had much experience in managing that disease. It frequently determines the crisis, and leads to convalescence, with improvement in all the symptoms.

It fulfills the most important indication in the treatment of inflammatory disease, namely, by relieving the kidneys of much of their work. It "puts the parts largely at rest," and permits the impeded or exhausted forces to regain their tone. Thus, the skin should be subjected to the most systematic care in all forms of albuminuria—no less so, in fact, than in the treatment of phthisis. Chills to the skin should be most carefully guarded against, as they lead to hyperæmia of the kidney resulting from internal congestion, which always follows contraction of the cutaneous vessels. Besides this, when the action of the skin is checked there is greater accumulation of nitrogenous waste in the circulation, which, as we have seen, is another cause of hyperæmia. Warm flannel undergarments must be insisted upon from head to foot, from which no variation must be permitted; even in bed flannel night dresses must be worn. The care of the feet must be no less rigid; heavy soled shoes and boots should always be worn, lined preferably with some warm, non-conducting substance, as cork or felt. The patient should be taught to follow carefully all changes of climate with appropriate external clothing and wraps, and all garments becoming damp from exposure should immediately be changed for dry and well warmed fabrics, and this is doubly important in case of the feet and extremities. There is no more constant or frequent cause of nephritis than exposure to cold; and when the vital powers are lowered, as they are usually in all forms of albuminuria if of any continuance, the susceptibility of the patient to the pernicious effects of cold are very much increased. The depressed, partly paralyzed vasomotor nerves of the skin leaves the cutaneous system almost at the mercy of sudden changes of temperature; hence are requisite the most rigid precautions to prevent exposure under these circumstances. It may be, and indeed it most frequently is the case, that the kidney is not damaged in its entirety; but parts of the organ continue to exert their function compatible with the continuance of life, perhaps indefinitely so long as the lesions do not extend. It therefore becomes of no less importance to guard against causes which are likely to light up an extension of the disease to the healthy structures, than it is to arrest the morbid process in the parts already invaded; and in no way may such objects be more subserved than in the care of the skin through proper attention to clothing, and the avoidance of exposure to vicissitudes of climate. It is proper that we here allude to the general influences of climate on Bright's disease. The first thing to be noticed, is the fact that in the two extremes of temperature—the arctic and torrid zones—albuminuria is rare. In extremely cold climates the great activity of the respiratory system takes the place of diminished action of the cutaneous system, a compensation which relieves largely the kidneys of the extra work which would otherwise be thrown upon them. Consequently we find the greater tendency to diseases of the air passages and respiratory organs under such circumstances. In the tropics, on the other hand, we find the greatest activity of function in the cutaneous system and bowels, and as a consequence the tendency to disease is more largely to these organs. The kidneys are but little taxed in the tropics, and Bright's disease there is almost unknown. We must make one exception to this statement in the case of amyloid disease, which sometimes does arise in hot climates. It must be remembered, however, that amyloid disease is an affection whose primary seat is almost never in the kidneys; and moreover, it owes its origin to well known constitutional causes, with which the kidney is not primarily concerned. The temperate zone, then, especially in locations proximate to large bodies of water, where changes of temperature are frequent and extreme, is the natural home of Bright's disease.

Whether through the effects of constant cooling of the skin, through the influence of the cool, moist atmosphere of winter, or the exposure to sudden changes which we find so common under these conditions, we get in the first place a gradual leading up to interstitial change, or in the other the sudden precipitation of nephritis, which seldom occurs either in the tropics or in the land of snow and ice. The climate most suitable in these cases should be equable, warm, and, if possible, dry. Dickinson says the mean temperature for the year should be between 60° and 70° F. for a patient suffering from Bright's disease. He supports the assertion by a tabular statement showing the mortality from Bright's disease in the British army in the various stations over the world, as well as in the larger cities. He shows, for instance, that in some of the cities of England and Scotland the mortality from Bright's disease reaches as high as 1 in 200, while in Rome it does not exceed 1 in nearly 5,000, and in the Riveria it is unknown, so far as its origin there is concerned.

In America the southern interior of the continent presents the greatest advantages—such as Mexico. In Europe, the Mediterranean coast seems to give the best results; Rome, Cairo and the south of France may be mentioned in particular. Those engaged in practice near this chain of great inland lakes, where the climate is especially unfavorable, should send their patients to the southern interior, at least during winter, as it would prolong the lives of patients materially.

Nearly all forms of renal lesions, at some stage in the progress, are benefited by the use of diuretics. The proper selection of diuretics, however, is a matter of the utmost importance; for some of them, if administered inopportunely, without reference to the case, stage, or remedy itself, are capable of doing very serious mischief.

Diuretics may be classed under two heads, as to their mode of action—first, those that act by increasing the general blood pressure in the vessels; second, those that act locally on the kidney circulation and renal epithelium. Belonging to the first class are such remedies as Digitalis and Convallaria Majalis, which increase the power of the heart's action, and as a consequence cause greater fullness in the arterial vessels. Fluids, also, by increasing the volume of the circulating fluid in the vessels, increase the blood pressure and quicken the urinary secretion. Thus it is that most mineral waters act. To the second class belong such agents as juniper, buchu, and some of the resins. Their action is stimulating to the renal epithelium, and hence as a rule they are contraindicted in the acute inflammatory conditions; their judicious employment following the subsidence of the scute action is often productive of much benefit. Where a simple diuretic is indicated, devoid of stimulating action on the renal epithelium, waters as pure as obtainable are very serviceable. It would seem that the purer the water—that is to say, the less organic matter it contains—the more efficient is its diuretic powers. I find the administration of several glasses of distilled water daily to act efficiently in such cases I have been much pleased with the results which follow the use of Waukesha waters, when simple diuretics are indicated as above referred to. With the Silurian water I have had the most experience, and for general purposes of the kind I think it cannot be too highly commended, especially as we have it so near at hand. Carlsbad waters have considerable reputation in Europe. From some experience with the imported natural water as we get it here, as well as with the Sprudel salt, I have not found any special advantages.

It is a well known fact that the addition of alkalies and some neutral salts of the alkalies increase the diuretic powers of water, owing doubtless to their stimulating osmosis; and in the selection of mineral waters this fact is well to bear in mind. The alkaline properties of Vichy water commends it especially for use in cases of gouty history, which we find so frequently to precede the more chronic form of the disease, and the Buffalo Lethia waters are even more valuable in such cases.

ACUTE PARENCHYMATOUS NEPHRITIS—Whatever differences of opinion may obtain in other renal lesions as to their true pathology, there can be no question as to acute nephritis. The process is distinctly inflammatory, and must be met with energetic antiphlogistics. The more early this fact is recognized and the more promptly measures are shaped accordingly, the more successful will be the result of its management.

Two prominent indications present themselves in dealing with acute nephritis. First, to secure depuriation of the blood through stimulating the action of the skin and alimentary canal; and second, to limit the inflammatory process in progress in the renal tissues. The most efficient means of exciting the action of the skin in these cases is the *dry hot air bath.* The steam or Russian bath, or the baths devised by Simpson, nor yet the hot water bath in these cases must not be ranked in efficiency with that of dry hot air. There should be no mistake on this point. Though it may be a more convenient method to wrap the patient up in hot, moist blankets and leave him to steam for an hour or two, the results will not be so satisfactory in producing elimination of fluids by the skin, and moreover the practice is not so safe as the employment of dry hot air. The advantages of the latter are that evaporation takes place more rapidly in dry than in moist air, hence more rapid perspiration is induced. Again, the bodily temperature is not elevated near so high with dry hot air as by means of steam for the heat of the body is obstructed in its egress and accumulates more rapidly than in dry air. I have induced the most copious perspiration for an hour or more by means of the hot air bath, without elevating the bodily temperature more than one and a half to two degrees, and it seldom raises the temperature more than the latter. It is well known that the Russian bath, so-called, usually increases the bodily temperature three to five degrees, the pulse is accelerated to 150 or even higher, with a corresponding effect on the respiration. Should the case be complicated with heart trouble, such disturbance of the system is highly injudicious, and if the patient is threatened with uremia the great increase of temperature is more apt to precipitate convulsions. In all cases, then, the dry hot air bath is preferable; it promotes a more thorough action of the skin, without seriously disturbing the pulse, respiration, or bodily temperature, and moreover, it is far more comfortable to the feelings of the patient. The application of the hot air bath is not necessarily attended by more inconvenience than the hot pack, much less the hot water bath. The simplest contrivances often meet the necesties of the case quite as well as all the paraphanalia of the modern Turkish baths. The patient may be placed between woolen blankets, carefully tucked in about the edges of the bed and the neck, leaving the head out of course.

Plenty of heavy quilts are next thrown over the patient to retain the heat. A small alcohol pocket lamp is next filled and placed on a tray near the bed, fitting over which a small sized stove pipe rising about three feet from the floor conducts the heat beneath the blankets beside the patient. To secure a more equable distribution of heat about the patient an oval crib formed by joining a few half barrel hoops with half a dozen laths, may be placed over the patient which also serves to keep the blankets from weighing heavily on his body, and secures quite a roomy air chamber beneath the bedclothes. By such means a patient may be subjected to free diaphoresis for an hour or two with the least attendant exhaustion possible. The patient need not be removed from his bed—an important consideration if there is much prostration. I do not claim any originality for the above method of employing the hot air bath; but I do claim advantages for its use over any and all methods of producing diaphoresis with which I possess any knowledge. In cases of acute nephritis it should be employed daily for an hour or longer, according to the urgency of the symptoms and the strength of the patient. It will be found after the patient is made to perspire for half an hour or so, that the artificial heat may be stopped, and perspiration will continue freely for a long time, more especially if some warm drink is administered. He may then be quickly dried with warm towels and comfortably arranged in bed. Jaborandi, or its active principle, pilocarpine, may be tried and will sometimes act very freely as a diaphoretic. Its uncertainty, however, and occasionally its depressing effects render the hot air most to be relied on. Free catharsis should next claim attention, and the selection of the purgative will be determined by the strength and age of the patient as well as the urgency of the symptoms. While not advocating a continuous course of purgatives for the relief of nephritis, we favor beginning treatment with a brisk purge, which besides in itself beneficial, it very much increases the action of other remedies, for by unloading congested blood vessels, it is well known that it relieves stasis of blood and promotes osmosis and absorption. As a rule we should begin with free catharsis, then regulating the bowels subsequently with milder aperients as the most conservative course.

In addressing our treatment to the kidneys themselves, all authors are agreed that dry cupping is highly beneficial. The cups should be quickly moved when the skin becomes reddened, and the process may be repeated several times during the day. Following the cupping large warm poultices should be applied to the lumbar region continuously, and their efficiency may be much increased by adding sufficient mustard to keep the skin irritated. In a later stage more permanent and decided counter irritation is beneficial, as by the use of croton oil liniment, but blisters of fly, or turpentine are on no account to be employed for obvious reasons.

The question of the use of diuretics in acute nephritis is one which has been warmly disputed, and calls for most careful consideration. Dickinson does not sanction their use unless it be in the form of spring water. Even with regard to digitalis he says : "I have learned to doubt whether this sure diuretic is so certain not to add to the congestion of a recently inflamed kidney, as I formerly supposed. It adds to the force of the heart and I think I have sometimes traced the discharge of blood with the urine to its use."

Grainger Stewart, on the contrary, says : "I have been accustomed since student days to see diuretics freely employed and frequently with great advantage." Roberts claims for the exhibition of acetate of potass a uniform increase in the quantity of urine, a subsidence of inflammatory action, and a subsequent exemption from uræmic attacks.

In examining this question critically, it may be useful to remember the method whereby nature determines the crisis in cases which take on a favorable course. If, for instance, a case of nephritis is left to itself and no medication employed, either complete blocking up of the renal tubules will occur, and death follow from dropsy, or uræmia, or spontaneous convalescence will follow upon most copious diuresis. Nature's most potent avenue of relief then is clearly through diuresis. If we can bring about this result without increasing the existing damage to the kidneys themselves, the agents which will accomplish this are clearly those upon which we should rely. Bearing in mind the pathology of the lesion we seek to remedy, the secreting tubules are blocked up with various products of diseased or disorganized epithelium which line these tubes. Stagnation in the packed tubules prevents filtration of urine, and there can be no improvement till the obstruction be removed. Obviously, if we use such diuretics as juniper or squill, which stimulate the renal epithelium, we run the risk, not only of adding to the renal congestion, but also of hastening the disorganization of renal epithelium, which would increase rather than diminish the blocking up of the tubules. Fortunately, we have a class of diuretics which quicken the filtration of urine in the malpighian coils, through their power of augmenting the general blood pressure without any stimulation of the tubular epithelium. Agents which directly increase the power of the heart, of which digitalis is the representative, are the ones indicated. Fluids also, such as water and salines, lead to the same result as heretofore pointed out. The aqueous elements of the urine are in the main filtered through the malpighian coil, an arrangement peculiarly fitted for that purpose; increase of blood pressure then results in a more copious filtration of urine within the glomerule. The urine is forced downward washing before it the obstructing débris, or products of inflammatory action, without exerting a deleterious action on the tubules themselves or their lining epithelium. We believe, then, that a judicious selection of the diuretics referred to are among the most efficient agents at our command. Such are the measures in the main most useful in the management of acute parenchymatous nephritis; and it is not too much to assert that by far the larger majority of cases managed in accordance with the principles dwelt upon will terminate favorably.

It is scarcely necessary to add to what has been said already on the question of diet, except perhaps to say that the most rigid adherence to the rules laid down before, should especially be carried out in acute nephritis. Milk diet, more or less varied, should be adhered to, to the exclusion of meats. The patient should be confined to the bed from the start, and kept there until the acute symptoms have quite passed away.

Complications of several kinds are apt to accompany acute nephritis. The most alarming of these is uræmia, which in this lesion is most apt to assume the tonic convulsive form. The physician should ever be on the watch for this in treating nephritis, for its appearance is usually preceded by well defined premonitory symptoms, which should be taken advantage of in time. If the urine diminishes in quantity to four or six ounces in twenty-four hours, we know that much waste material is accumulating in the blood. The urine may even be fairly voluminous, but containing daily a minimum of urea, and this we know is apt to lead to a dangerous accumulation in the circulation. Possibly vomiting may occur and become obstinate, and more or less headache, and dullness or drowsiness supervene. Symptoms like these must be met by prompt measures, as they portend the near approach of convulsions. The hot air bath should be resorted to, and its use prolonged till the most copious diaphoresis is secured; following which a sharp purge should be administered. Among the agents advised for the uræmic attack itself are bromide of potash, chloral hydrate, chloroform, and hypodermics of morphia. The latter we should hesitate to employ, though Prof. Loomis, of New York, and even Ringer, indorse it. We should administer full doses of chloral hydrate, either by the rectum or hypodermically, and if the convulsions were still persistent, should resort to venesection, at least as a temporary measure. It has been well known for many years that bleeding gave the best results in cases of puerperal convulsions; even before the cause was understood. We now know them to be uræmic, and if bleeding will arrest the uræmic convulsions of pregnancy, there appears no reason why it should not likewise be equally serviceable in similar eclampsia of nephritis.

Acute chest affections, when unfortunately they complicate nephritis, constitute a class of troubles the most difficult to manage. Their intractability is due to the fact that they are induced by a poisoned condition of blood, incapable of furnishing the elements conservative of tissue destruction ; as well as being incapable of removing the products of inflammatory change. To the usual antiphlogistic measures employed in these inflammations, must be supplemented the energetic use of all the measures tending to eliminate, through the various compensating channels, the retained tissue waste circulating in the blood ; and these have been fully dwelt upon heretofore.

Dropsy frequently constitutes one of the most obstinate symptoms of acute nephritis ; and occasionally becomes so extreme as to call for special measures for its relief. Its appearance signifies that the aqueous elements taken into the system are obstructed in their elimination. In other words, it points, as a rule, to grave obstruction in the secreting structure of the organs. Sooner or later, if the organs do not resume their function, the dropsy calls for relief, else crowding of the organs within the chest may lead to death through gradual asphyxia, or impeding of the heart's action ; or local erysipelas or gangrene may be set up through compression of tracts of the surface, usually of the limbs. Puncture of the skin in places will relieve the tension of the parts, but such measures often lead to very troublesome ulcerations and sloughs, and, as a rule, are to be avoided.

There is less objection to draining away the surplus fluids from the abdominal cavity, or even the chest, by means of the trochar or aspirator ; and this may be done as a temporary measurse, often affording great relief to the patient. It must not be lost sight of in this, as in all complications of nephritis, that the only permanent relief must come through a re-establishment of the renal function ; and while special means are being taken for the relief of the various complications as they arise, the general measures laid down for the improving of the kidneys themselves must at all times be resorted to with a firm hand. If this be neglected, any seeming success in relieving a threatening complication is only followed by the greater misfortune of discovering later a general condition which will baffle all our efforts to relieve. If dropsy become urgent, and the general condition of the patient is not much reduced, we may often obtain good results from evacuent treatment. In such cases American hemp may be used often with the happiest results. It should be given in repeated doses, till nausea is induced, and then its use suspended for a few days. The diuresis produced by its action is sometimes enormous. From a few ounces of dark red, smoky urine, passed in twenty-four hours I have known it to be immediately increased to several pints in a few hours, and the dropsy to disappear in two or three days. Sometimes it acts upon the bowels as well, producing copious watery stools. If, fortunately, free diuresis is once established, its continuance may usually be kept up by the other measures advised; and if so, the patient enters upon convalescence. Chronic nephritis should claim some attention before speaking of the other renal lesions. Rarely beginning as such, chronic nephritis is usually the result of a more or less prolonged acute attack. The inflammatory action having largely subsided, the tubular epithelium remains more or less damaged, with a tendency to fatty degeneration. Pretty much the same general principles should be observed in treating the chronic form of nephritis as the acute, except that greater caution is necessary in the employment of evacuants, as the general condition of the patient is usually reduced. Purgatives, for instance, should not be pushed, as they are likely to increase the anæmia so frequently present.

Bartels lays great stress on diaphoretic treatment. He says: "The methodical employment of diaphoresis constitutes, in my opinion, the most reliable means of reducing the troublesome and dangerous dropsy; it is also the only treatment from which I believe myself justified in expecting a curative action similar to that which it exerts on the acute parenchymatous inflammation of the organs."

We must bear in mind that in the chronic form we are dealing with a deep-seated malady, whose changes are less active than the acute form, and any measures employed must be continued systematically over much longer intervals of time in order to secure any permanent results. Diaphoresis, if conducted with these facts in view, through the agency of hot air baths, in keeping with the strength of the patient, will seldom disappoint our expectations unless the disease has progressed so far that resolution is impossible. I have used them daily for weeks without obtaining the exhausting effects attributed to them, and never without the most marked improvement in most of the symptoms of disease. Thick woolen textures should be worn next theskin, which not only insure warmth of the skin and invite the blood to the surface, but also absorb the prespiration and prevent the sudden check of the latter by changes of temperature. We are not as closely restricted in the use of diuretics in this as in the acute form of the disease. The free washing out of the tubules is most important as it promotes a more healthy action of the renal epitheluim, and tends to restore the damaged condition of the gland. Some of the spring waters already mentioned may be taken, say three to six glasses daily. The citrate or the acetate of potash are very favorable diuretics, as they are in the acute form. Whichever of these salts are employed they should be given in full doses. As a rule, the more recent the case, the more may be expected from the use of these salts, though they often act happily also on cases of long standing. If the urine persists in scantiness, containing considerable dèbris, it is often beneficial to employ some of the diuretics which are more directly stimulating to the kidneys themselves, as the contra-indications to their use are not so strong here as in the acute form. They are preferably combined with some of the potash salts. Juniper and squill are of doubtful utility unless it should be in cases of great chronicity. I use frequently in these cases a combination consisting of compound fluid extract of buchu and pareira brava with acetate of potash. Most cases of chronic nephritis, sooner or later, require the use of iron on account of advancing anæmia. It is best to combine with the iron, or give at the same time, some alkalie salt. I believe it was Trousseau who first pointed out the advantages of this in all cases of anæmia. The beneficial effects of a warm dry climate mus tnot be lost sight of. If the patient can afford to avail himself of such valuable aid to treatment, it should be strongly advised above all during winter months.

*Granular Degeneration.*—It must be conceded, I think, that when the kidneys become the seat of interstitial fibroid growth to any serious extent, it constitutes a condition from which recovery of the damaged organs is well nigh hopeless. The fibrous tissue growth not only impossible to reach and remove by remedies, likewise causes destruction of the tubules so complete that restoration is not within the reach of therapeutics as at present known.

It would seem from this at first sight, that the outlook of a case established as renal fibrosis must be a hopeless one. Fortunately, this is not absolutely so, as far as prolonging life, and even arrest of the disease is concerned. Granular degeneration of the kidneys, as we have previously observed, is the most tardy and chronic in its course of all kidney diseases, extending sometimes over fifteen or twenty years, under favorable circumstances, ere the entire organ becomes sufficiently implicated to put a stop to its function or impede the same incompatibly with the continuance of life. Dickinson remarks, "The organ is invaded by slow, and often hesitating approaches, and we cannot but admit the importance of any measures which, by removing the tendency to the disease, may save as much of the gland as is yet intact."

When we reflect that the great majority of these patients are beyond middle age, in fact well advanced in life, and the symptoms are not necessarily incompatible with a fair degree of enjoyment of life, the outlook seems less gloomy. Probably the greatest influence capable of being brought to bear in limiting or even arresting the disease, may be looked for in climate. An even temperature, with a mean between 60° and 70° F., with a dry atmosphere, are the desired objects to be attained. Dickinson has paid much attention to the influence of climate over granular kidneys. He says, "I have sent persons with whom the disorder, though giving rise to no symptoms which could unfit them for traveling, yet has been advanced and threatening, to winter in the Riveira, the warmer parts of Italy, or Egypt, with the issue of early general amendment, and such certain though slow mitigation in the special symptoms as to show that the advantage to be expected from change of sky is at least as great in renal as in pulmonary disease. Cure is a word to be used with caution, but I have seen little less: the albumen reduced to a trace, and perhaps that inconstant, and the general health brought up almost to its original level."

If the disease is fortunately discovered in its early stage, then the patient should be sent to some such climate as before indicated. Rome is spoken very highly of, as is Cairo. The disease is almost unknown in the former place. I am not aware what results have been recorded from sending patients to the more favored climates of our own Continent. I believe the Mexican climate to offer the most desirable conditions for residence. Take, for instance, Durango, where the extremes of temperature are 60° and 80°; the atmosphere is dry, and the elevation is about six thousand feet above the sea, sufficient to keep the respiratory functions in a state of activity. Aside from the months of July, August and September, which constitute the rainy season, it affords superior advantages for this class of patients. In a disease so impregnabie to the effects of medication we must rely more on the influences of climate, which in this case fortunately often proves potent in retarding, if not arresting, the disease.

As to the use of medicine in granular degeneration of the kidneys, they will be found more potent in protecting the patient from the effects of complications than upon the disease *per se.* We must have regard to the fact that this lesion is for the most part led up to through an increased activity of the gland which has been long overtaxed, resulting at length through continued functional hyperæmia in proliferation of inter-tubular fibration. The gland may suffer in isolated sections, some of which may come to a stand-still, or other new points may develop and continue independently and thus, step by step, the whole gland gradually succumbs, not by a general process simultaneously through its whole corticle at once as in nephritis. The lessons to be learned from this are, that if we can modify the conditions which give rise to the morbid process, we have greater chances of checking its progress than would at first be expected. Advantage may be taken of the temporary lull in the progress of the disease so common, to place the patient under conditions less likely to favor its subsequent progress.

Remembering how Bright's disease is largely resultant from indulgence in too highly nitrogenized food as a habit, the diet must be regulated to contain barely sufficient of such to maintain tissue repair. We have already laid down the rules which should govern such cases, and it is only necessary to add here that they must be still more rigidly adhered to in this lesion if the patient's life is to be preserved. The careful regulation of the diet, and also of the condition of the skin, constitute the two chief points on which depend aggravation of the existing lesion, and no less so the determination of serious complications. By keeping the skin warm and dry, and lessening the nitrogenous waste in the blood, we avoid with most certainty the acute conditions which sometimes arise; and be it remembered, that in these acute conditions is where the chief danger to life lies. All that we have said under the head of nephritis with reference to clothing, is quite as applicable here, and need not be again gone over.

By referring to a table of urine in my former paper on the pathology of Bright's disease, published in this journal for April of this year, it will be observed that the average quantity of urea excreted by the kidneys daily in renal fibrosis, is 160 to 280 grains; consequently, from 232 to 352 grains of urea more than the normal is retained daily in the circulation, either as urea or the products from which urea is formed. Now, herein lies one of the great dangers to be apprehended in granular degeneration. As a result of the above, uræmia often explodes suddenly; or, almost equally serious, the lighting up of some acute inflammation within the chest, which is even more intractible than uræmia itself. It will not do to await the onset of such complications as these. All rational treatment of renal fibrosis must have constantly in view the threatening of such dangers, and measures must be constantly employed from the first which are calculated to avert the mischief. We have referred to the necessity of diminishing the nitrogenous, and increasing the carbo-hydrate foods. Further measures will be the constant maintenance of the bowels in a regular condition. Occasional hot air baths are of immense importance in securing free elimination through the skin, of waste products. The kidneys are, perhaps, on the whole, best kept active by means of alkali salts; but heart tonics, as digitalis, I believe are unsafe, and should be limited at least to special cases, where, for instance, there is no evidence of over-tension in the vascular system. The reason for this caution lies in the fact that a large proportion of the cases of renal fibrosis terminate in cerebral apoplexy. It is believed, with degeneration of the arterial vessels and hypertrophy of the left heart, so constant an accompaniment of this lesion, that the employment of heart stimulants constitute no ordinary danger, which should be restricted, not only as to drugs, but also in the avoidance of all muscular exertion which calls suddenly or heavily upon the heart's action.

Various drugs have been recommended as exercising a direct curative action on the kidney itself. Perhaps the most popular of these are iodide of potassium and the chloride of gold; but the records as yet are not sufficiently encouraging to inspire great confidence in their use. Iodide of potassium theoretically promises the best results, and some authors recommend it strongly. Bartels gives the drug to the extent of 20 to 30 grs. daily, continued for an indefinite period. He says in one of the cases treated for four months thus, "the albumen had as good as disappeared from the urine; the amonnt of urine fell from 2,000 c.c. on his entrance to the hospital, to 1,400 c.c. on an average." Too much reliance should not be placed upon such measures in the light of past experience. Bartels himself adds: "The patients whom I treated upon this principle, before threatening symptoms appeared, withdrew themselves from my observation too early, so that I cannot yet venture to form an estimate of th ultimate results of this method of treatment." As to chloride of gold, I am unable to say that it has any influence in checking the disease; but its use should be extended over greater length of time than I have had opportunities of observing its actions, but aside from its tonic action on the stomach, uniformly improving the appetite as it does, I should not be inclined to have much faith in its powers over the disease.

Speaking generally of the management of this disease, sometimes during the history, evidences of high blood pressure are apt to be present—as dizziness, palpitation of a powerful nature, heaving the chest-wall visibly over larger space than usual, oppressson of the respiration, full, tense pulse, etc. Such symptoms should be met by rest, low diet, cardiac sedatives, and in extreme cases by venesection. In the latter stages of the disease we are likely to meet with somewhat opposite conditions of debility and anæmia; and these should be met by tonic suppurative measures, both general and special, and even with stimulants. Iron may be given as in the chronic stages of nephritis to counteract advancing anæmia. If the heart's action weakens, as it sometimes does in this stage, we know that it must be maintained, else the remaining portion of the gland will fall functionally below the conditions compatible with maintenance of life. Good Burgundy, or other red wine, should be allowed, as well as a more stimulating diet. It may even be advisable to administer digitalis in small doses here, and often with excellent results, as seen in the removal of dropsy. Sometimes complications threaten the patient from another source—as the lighting up of nephritis in addition to interstitial disease. Some increase of temperature, diminished urine, containing epithelial casts, higher specific gravity of same, and a larger amount of albumen mark the approach of nephritis. The measures advised before for nephritis are to be employed and pushed with corresponding activity, to the demands of the case.

Such are the general principles which we believe to be the safest guides in the management of granular degeneration of the kidneys. It is surprising, sometimes, to see the apparent health with which some patients progress for years, if carefully guarded against complications, even after very threatening symptoms have made their appearance. I append from my notes case 32, first seen September 3, 1882. Patient is 64 years of age; gouty history; had several attacks of vertigo within the past year; fell to the ground several times as a result thereof. Urine, 50 ounces daily, sp. gr. 1010; contains 10 p.c. albumen, and a few pale casts. There is slight œdema of feet; great itching of the skin, with erythematous rash. Headache and drowsiness are prominent. Radial pulse very tense and full; the carotids pulsate visibly. Complains of fullness in the head. The heart's impulse strong, with extended area of dullness. Patient is weak, restless, and odor of urine is perceptible in the breath. He has been too much prostrated to be able to walk for weeks past. October 20, 1883, over a year after patient came undor my care, condition is as follows: Patient has gained in flesh during past year something over twenty pounds; is able to walk about, appetite is good, sleeps well at night, is clear in intellect and lively in spirits. He suffers no pain. In short, he enjoys life equal to the average, in every respect, of men his age. The urine is about normal in quantity, contains about 6 p.c. of albumen and a few pale casts. It is with difficulty I can make the patient realize that he is an invalid; yet it is plain that over a year ago he was on the brink of uremia and apoplexy, the latter of which caused the death of two of his family.

In closing the consideration of renal fibrosis, we wish to emphasize the fact, that success in its management will depend largely on its early discovery. Creeping on stealthily, as it always does, it will elude observation, unless the utmost watchfulness is exercised. No dropsy or abnormal condition of the urine is likely to attract our attention, or that of the patient, to the nature of the disease. The constitutional or special physical peculiarities of the patient himself must often be the sole guide to investigation. An unusually full pulse, with other evidences of vascular tension, should be noted. A gouty habit must not pass unobserved. Method of life should be noted, particularly as to indulgence in the pleasures of the table. By habitually noticing such points as these in our patients, we get to know what class of cases we are most likely to find fibrosis developed from; and thus, by anticipating the approach of disease, we make its discovery more frequent in the early stages, the advantages of which cannot be overestimated.

AMYLOID DISEASE.

In the special management of this lesion, we should have in view the fact, that it essentially differs from the preceding forms of disease in its nature, and requires an entirely different method of treatment. In nephritis and fibrosis, many of the principles underlying the treatment are very similar, and the management corresponds accordingly, often varying only, in the main, with the activity with which the measures should be employed. Both of the preceding diseases begin in the kidney itself, and though pursuing a different course, owing to the processes attacking different tissues of the same gland, yet the force of both is ultimately made known through serious interference with the function of the gland, and measures must be largely directed in both to the restoration of that loss of function. But in amyloid disease the case is quite different. In the first place, the amyloid kidney constitutes only a part of the chain of morbid processes whose origin and cause are quite outside of the kidneys themselves, and our measures will be more largely directed to these outside influences which give rise to the disease, than the measures directed to the lesion in the renal structures themselves. In the second place, the function of the kidneys is far less interfered with here, than in the lesions already considered; and we seldom have to contend against the dangers of uræmia, or like complitions. The patient is permitted to live the natural duration of the disease, unless, in the advanced stages, he succumbs to diarrhœa or dropsy. In the third place, the disease carries with it from the beginning the stamp of debility and cachexia, and calls for active supportive measures, in all cases and in all stages. It is fortunate that the amyloid kidney performs fairly its function till very late in the disease, as it permits us to carry out more fully this last indication. Urea is eliminated, little short of normal in amount, throughout the greater course of the disease, and, as a consequence, uræmia need seldom be feared, save near the close of its course. We are thus enabled to resort to stronger meat diet in this, than in the other lesions, and the indications command us to do so with liberality.

From what has been said, it will be gleaned that preventive measures form a most important element in the management of amyloid disease. Syphilis, tuberculosis, chronic suppuration, in themselves largely under the influence of remedies, command our first attention, as the most certain method of arresting the resultant disease.

It is scarcely in keeping with facts to rank this disease in the list of incurable maladies, and deprive the patient of hope, as has been the habit with some. Amyloid disease is at least as curable as the causes which gave birth to it, and as these are removed, the progress of amyloid degeneration will usually cease; and not only so, but the lesions caused thereby themselves are capable of being removed. He would be rash indeed, in the presence of past experience and present facts, who would affirm that phthisis, chronic abscess, necrosis, dysentery, and above all, syphilis, are incurable; yet these are the most prolific causes of lardaceous kidneys, and the progress of the latter will depend on the control of the former.

Generally considered, all these patients require tonics, nourishing diet, and all measures within our resources which will improve the constitutional debility. The condition of the blood calls for the use of iron, which must be continued over an extended length of time. The neutral preparations of iron are better borne, owing to the constant weakness of the stomach in those cases. Blancard's pill is also a peculiarly appropriate form, consisting of iodide of iron, well preserved through the peculiar mode by which it is prepared. Food should be the most nourishing possible, but the most easily assimilable at the same time, should be selected, in order not to precipitate diarrhœa, so frequently the accompaniment of this disease. Red wine and diluted spirits are often great aids to nutrition, and besides, tend to stay the general tissue waste, so marked a character of this malady. Tubercolosis, perhaps the most fruitful source, requires to be treated in accordance with the principles most successful according to recorded experience. It is to be hoped that the recent important discoveries in the pathology of tubercular disease by Koch, may lead not only to more successful treatment of the same, but also, as has been suspected already by some, to the possibility of an important rôle which bacteria may play in the disease under consideration. In the management of the various suppurative processes which give rise to amyloid disease, the question becomes one largely for surgical measures. It is a fact upon record, that in those hospitals where Listerism has been most thoroughly carried out, the number of amyloid cases have very strikingly diminished. This lends strength to the rule that where suppuration exists in chronic form, more especially in bone, it must, if possible, be controlled, even at the expense of sacrificing a limb. A patient can never be considered safe if a necrosis continues, though perhaps not in itself producing much drain on the general health. The urine should be examined frequently in such cases, and on the appearance of albumen operative measures must at once be employed, if practicable. I treated a case of amyloid disease of the kidneys, with which some of you are familiar, resulting from necrosis of the right humerus, which illustrates the length of time suppuration may continue without apparent damage, and considered harmless, perhaps, by the patient, till some other disturbing cause of health precipitates the amyloid degeneration. In the case referred to, the necrosis continued for twenty-two years before any kidney lesion was developed. Sometimes we meet with the amyloid disease, resulting from suppuration, where an operation may be inadvisable, the patient's general condition not warranting such risk. In all such cases it is wise to resort to the minutiæ of Listerism, for the reason that it practically isolates the destructive action from the atmosphere. As a rule, so long as suppuration is limited within a walled cavity it does not give rise to amyloid disease, but when a communication becomes established with the surface, and atmosphere gains access, the mischief ensues to the kidneys.

By antiseptic dressings we may sometimes practically silence the provocative of the disease, and in the meantime by tonic measures improved the general condition of the patient to a point where an operation would become admissible and thus possibly save the life of the patient, where otherwise he must necessarily have succumbed. When the disease is traceable to syphilis, specific medication will often be followed by most satisfactory results. With the minutiæ of this it does not fall within our province here to particularize, especially as the treatment of syphilis is of late clearly defined in excellent special works. Although amyloid disease is usually the sequence of the tertiary forms of syphilis, and iodide of potassium may be used with great confidence in relieving the symptoms, we may say that the prevailing opinion is in favor of the use of minute doses of mercury long continued, after the method of Dr. Keyes, of New York, not to the exclusion of iodides, perhaps, but in combination therewith. The iodide of potassium is recommended by Bartels in all cases of amyloid disease which seems to be the only medicine he employs with the view of attacking the disease itself. It is doubtful, however, if its use is attended by any striking benefits, unless in those cases which arise from syphilis. Not so, however, the combinations of iodine, as with iron, for instance, which is a most desirable form of tonic alterative. Dickinson speaks of the use of alkalies, especially in cases attended by purulent loss. His object seems to be with the view of making good the loss of alkalies, always so great in suppuration; and theoretically also because amyloid matter is so soluble in alkalies. He afterwards admits, however, that "disease cannot be treated in a test tube," and goes on to explain that the alkalies are well borne, and in connection with other tonics give better results than tonics alone, but that on the whole "their use is disappointing." Sooner or later we shall be called upon to contend with the most obstinate and serious of the complications attending this disease, which directly or indirectly is the most frequent cause of death namely—diarrhœa. For the successful management of this complication it is well to bear in mind the pathological cause, and this is due to the degeneration of the small intestinal arteries, the epithelium of the mucous membrane and the muscular coat of the villi. The number of remedies recommended for this obstinate affection is quite formidable, and covers nearly the whole vocabulary employed for the relief of intestinal fluxes. The preference should be given to sulphate of copper in one-eighth to one-fourth grain doees combined with some opiate as the compound soap pill B. P. But if the stomach does not bear the opiate well it is better to to give it by the rectum preferably in the form of deoderated tincture. The accompanying dyspepsia is to be managed as in similar conditions elsewhere found.

Dropsy seldom complicates the case till the latter stages are reached, and then it is less within the range of relief than in other forms of kidney lesions. We may relieve it by means of hot air baths, but it soon returns owing to the impoverished condition of the blood, and extreme damage to the glands, as well as the degeneration of the small vessels. We must use the hot air bath more cautiously here, owing to the greater exhaustion of the patient. Stimulants and tonics containing iron are apt to give more permanent relief to dropsy here than the evacuant treatment. Diuretics as a rule are not called for, as the urine is largely above the normal in quantity. Visceral complications are apt to make their appearance as enlargement of the spleen and liver, which sometimes becomes enormous. The nature of the disease is the same here as in the kidneys, in fact, a part of the same process of degeneration of blood vessels and deposit of the peculiar amyloid material. If special measures become necessary for relief of visceral enlargement, perhaps the best agents are iodides, stychnia and hydrochlorate of ammonia. Uræmia, as before intimated, seldom appears, save in the very last stages, and then tonic convulsions are the exception; slowly approaching stupor followed by coma closes the scene. Throughout the course of amyloid disease we should bear in mind that, as in fibrosis, we may have nephritis engrafted upon the case at any period of its history. The early invasion of such complication it is of importance to discover and apply the appropriate treatment therefor. We shall not be able to push the evacuant treatment here so energetically or so contiuously as in simple nephritis, as the patient's general condition will not admit of lowering measures; hence the most careful adjustment of remedies must be sought in order to reduce the force of the more active disease, and at the same time not to aggravate the progress of the more chronic lesion.

The completion of this paper fulfills a promise made a year since, to present in a series of articles the whole subject of organic renal diseases; their pathology, diagnosis and treatment. In a field so prolific and varied as to its literature, the question which we have had to solve has not been what we should say, but rather what we should not say. Few subjects in medicine are so replete in literature, and at the same time on which opinions have been so unsettled, in nature, pathogenesis and therapeutics as this question of Bright's disease. Few diseases possess so extensive a list of remedies put forward with claims for its relief, which only tends strongly to the conviction that few of them are worthy of confidence. We conceive it to be of equal importance under such circumstances to eliminate those agents of doubtful utility as it is to indorse those which possess substantial merits. This paper is therefore burdened with the mention of few drugs and fewer formulæ, as we believe a proper undrestanding of the therapeutical indications of the diseased processes will lead to the most judicious selection of remedies in each and all cases. The principles involved in the treatment rather than the details have been our study to present, for once these are comprehended thoroughly, matters of detail are more safely left to the judgment of the therapeutist than the unscientific and uncertain vaunting of specifics. To succeed in the management of albuminuria one must cast aside all preconceived notions of specific medicines; the action of any such can never cover this wide and diversified field of morbid changes. The management must be reduced to philosophical reasons, as in the treatment of an inflammation, varying as it must when found in one class of textures, and again in another, and while endeavoring to fathom the true principles which should guide our management in these cases, we have endeavored to avoid the vast field of hypothetical speculation which has made the literature of this subject so voluminous and so uncertain, and have studied to draw rather from the record of clinical facts, than from the realm of theory the materials which we conceived would conduce more to the acquirement of practical knowledge available at the bedside of the patient, for after all, as Froude well says, "The knowledge which a man can use is the only real knowledge. * * * The rest hangs like dust about the brain, or dries up like rain drops off the stones."

68 Madison Street.

